# Cytotoxic, Cellular Antioxidant, and Antiglucuronidase Properties of the Ethanol Leaf Extract from *Bulbine asphodeloides*

**DOI:** 10.1155/2021/6622318

**Published:** 2021-04-14

**Authors:** Wilfred Otang-Mbeng, Idowu Jonas Sagbo

**Affiliations:** School of Biology and Environmental Sciences, University of Mpumalanga, Private Bag X11283, Mbombela 1200, South Africa

## Abstract

*Bulbine asphodeloides* (L.) Spreng (Xanthorrhoeaceae family), popularly known in South Africa as “ibhucu” or “Balsamkopieva,” is a perennial plant traditionally used to treat skin diseases, including sunburns, rough skin, dressing burns, itches, and aging. The present study reports the cytotoxic, cellular antioxidant, and antiglucuronidase properties of the ethanol leaf extract from *B. asphodeloides*. The cytotoxic effect of the plant extract on human dermal fibroblast (MRHF) cells was evaluated by the bis-Benzamide H 33342 trihydrochloride/propidium iodide (Hoechst 33342/PI) dual-staining method. A validated biological cell-based assay was used to determine the cellular antioxidant activity of the extract. The antiglucuronidase and metal chelating activities were evaluated using standard *in vitro* methods. Lipopolysaccharide- (LPS-) induced RAW 264.7 cell model was used to determine the anti-inflammatory effect of the plant extract, and the immune-modulatory activity was performed using RAW 264.7 cells. The extract demonstrated no cytotoxic effect towards the MRHF cells at all the tested concentrations. Furthermore, the extract also possessed significant cellular antioxidant and antiglucuronidase activities, but a weak effect of metal chelating activity in a dose-dependent manner. However, the extract showed no significant anti-inflammatory and immune-stimulatory activities. Overall, the results showed that *B. asphodeloides* may be a useful therapeutic agent for the treatment of skin diseases, therefore supporting its ethnomedicinal usage.

## 1. Introduction

Skin is more than a fleshy surface for tattoos, pimples, and wrinkles. It is the body's largest organ, with a total area of approximately 20 square feet, which plays a very significant function by protecting the body from microorganisms, helps regulate body temperature, and permits the sensations of pain, cold, heat, and touch. The skin is perhaps the most susceptible component of our body, and keeping healthy skin is important for a healthy body. It is a well-known fact that day-to-day exposure of human skin to the external environment leads to numerous skin diseases, such as sunburn marks, acne, and pigmentation [1]. In broad terms, skin diseases represent about 34% of all the diseases faced worldwide [2]. They are abundant and often occurring health problems affecting all ages from the neonates to the elderly and cause harm in several ways [3]. Skin diseases now occur as a main health encumbrance in both developed and developing countries. Despite the high frequency of skin diseases globally, mortality attributed to them so far has been relatively low, frequently persistent, and difficult to treat [4]. According to the World Health Organization, every year, approximately 180,000 people die from burns (fire-related burns) globally [5]. In South Africa, more than 19,500 burn-related deaths are reported every year, and they rank among the 15 leading causes of death among adolescents between the ages of 5–29 years [6]. This prevalence is driven by negative impact factors, including the inflow of people to urban areas, poor electrification of homes in low-income communities, and the use of paraffin and biomass fuels as the primary energy sources.

Human beings have been using natural resources empirically for skin care and also modifying their physical appearance. Medicinal plants have been extremely sought after to treat dermatological ailments due to their ability to treat burns, discontinue bleeding, accelerate wound healing, and lessen other skin conditions [7]. They are particularly significant and rich in antioxidant molecules that could impede oxidative stress or redox imbalances and alleviate the risk of emerging skin diseases [8]. However, the use of medicinal plants for dermatological inflictions has not been given the attention it so rightly deserves. This is astonishing as many plant species used traditionally for the treatment of skin conditions have not been adequately studied for their pharmacological efficacy.

*Bulbine asphodeloides* (L.) Spreng is a perennial herb from the family of Xanthorrhoeaceae (formerly Asphodelaceae), locally known as “Copaiva,” “intelezi,” “ibhucu,” or “Balsamkopieva” in South Africa [9, 10]. It is commonly used for the treatment of skin diseases in most parts of South Africa. Traditionally, the leaf gel is applied topically to wounds, sunburns, rough skin, and dressing burns [11, 12]. About 10 metabolites have been identified in *B. asphodeloides* by gas chromatography-mass spectrometry (GC-MS), and these were mostly sesquiterpenes (33.37%), fatty acid esters (12.62%), and diterpenes (8.03%) [13]. The phytochemical components identified from *B. asphodeloides* include anthraquinones and glycosides [14]. The root extract of *B asphodeloides* was reported to display weak inhibitory activity against *Erwinia carotovora*, a Gram-negative bacterium [15]. The ethnomedicinal uses of *B. asphodeloides* for the treatment of skin diseases provide justification for the undertaking of this present study to investigate the cytotoxic, cellular antioxidant and antiglucuronidase properties of the ethanol leaf extract from *B asphodeloides*. For this reason, this study set out to provide scientific understanding of its benefits and justification for its ethnomedicinal uses for the treatment of skin diseases.

## 2. Materials and Methods

### 2.1. Chemicals and Reagents

All the cell lines used in this study were purchased from Celonex (South Africa). The cell culture medium (DMEM, RPMI), phosphate-buffered saline (PBS), and trypsin were obtained from Hyclone, Logan, UT, USA. Fetal bovine serum (FBS) was obtained from Biowest (Logan, UT, USA). Hoechst, propidium iodide, and 2′,7′-dichlorofluorescin diacetate (DCFDA) were purchased from Sigma-Aldrich (St. Louis, MO, USA). The Annexin-FITC was obtained from MACS Miltenyi Biotec (Cologne, Germany), whereas enzymes, substrate, and other chemicals were obtained from Sigma-Aldrich (St. Louis, MO, USA).

### 2.2. Plant Material and Extraction

The fresh and mature *B. asphodeloides* leaves were collected in January 2020 from the Amatole district of the Eastern Cape province of South Africa. The voucher specimen (voucher no. BUR-2024) was authenticated and kept at the University of Fort Hare's Giffen Herbarium. For the extraction process, the powdered plant (30 g) was soaked in ethanol (500 mL) at room temperature for 24 h. Thereafter, the resulting mixture was filtered and then concentrated to dryness using a rotary evaporator (RVO 004; Ingos, Prague, Czech Republic) to obtain a dried extract (9.05% dry extract) [16].

### 2.3. Beta-Glucuronidase Assay

The test sample (10 *μ*L) and positive control, silymarin (48 *μ*g/mL), were pipetted into the wells of a 96-well plate. Then, 40 *μ*L enzyme solution was added to each well except those representing background and then incubated for 20 min at 37°C. Subsequently, 50 *μ*L substrate solution containing solubilize 7.88 mg *ß*-D-glucuronide-PNA (Sigma N1627) and 5 mL assay buffer (PBS containing 5% DMSO and 0.05% BSA) was added to the test well and background well, respectively, and then incubated for further 30 min at 37°C. After the incubation period, 5 *μ*L stop solution (0.1 M NaOH) was then added, and the absorbance was read at 410 nm.

### 2.4. Metal Ion Chelating Assay

The test sample (10 *μ*L) with various concentrations was mixed with 200 *μ*L deionised water, 20 *μ*L ferrous sulphate (FeSO_4_), and 40 *μ*L ferrozine. Thereafter, the resulting mixture was incubated at room temperature for 10 min, and the absorbance was read at 560 nm. EDTA (4 *μ*g/mL) was used as a positive control.

### 2.5. Cell Culture Condition

The human foreskin fibroblast (MRHF) cells were maintained in culture dishes (10 cm) in complete DMEM (low glucose) supplemented with 10% FBS, while the RAW 264.7 cells were maintained in culture dishes (10 cm) in RPMI supplemented with 10% FBS. All cell cultures were incubated at 37°C in a humidified atmosphere with 5% CO_2._

### 2.6. Cytotoxicity

The cytotoxic activity of *B. asphodeloides* on MRHF cells was assessed by the Hoechst 33342/PI dual-staining method [17]. Cells were seeded in a 96 well-plate at a density of 8000 cells/well using aliquots (100 *μ*L) and left to attach overnight. The treatment concentrations ranged from 25 to 100 *μ*g/mL for the plant extract. After incubation for 24 h at 37°C in a humidified incubator with 5% CO_2_, the treatment medium was removed and replaced with 50 *μ*L of staining solution (5 mL PBS with Ca^2+^ and Mg^2+^, 2 *μ*L Hoechst 33342 (10 mg/mL in DMSO), and 50 *μ*L Annexin V-FITC reagent) and incubated for 15 min at 37°C. Then, 50 *μ*L of propidium iodide (PI) (2 *μ*g/mL) was added, and the cells were then imaged using the ImageXpress Micro XLS Widefield High-Content Analysis System Molecular Devices (Molecular Devices®, San Jose, CA, USA). Nine image sites per well were acquired using FITC, DAPI, and Texas Red filters, and the images were analysed using MetaXpress® High-Content Image Acquisition and Analysis Software (Molecular Devices®, San Jose, CA, USA).

### 2.7. Cellular Antioxidant Assay

MRHF cells were seeded in a 96-well plate at a density of 8000 cells per well and allowed to reach complete confluence. Two days after confluence, the spent medium was removed by aspiration and then washed with Hank's Balanced Salt Solution (HBSS) to remove serum. Then, 50 *μ*L of the plant extract (12.5, 25, 50, 100, and 200 *μ*g/mL) or resveratrol (positive control) and 50 *μ*L DCFDA (25 *μ*M) were added. After incubation for 1 h at 37°C, the cells were washed twice with HBSS and then further washed with PBS. Thereafter, 100 *μ*L tert-butyl hydroperoxide (200 *μ*M) was added, and the fluorescence intensity was then measured at 485 nm (excitation) and 440 nm (emission) at 10 min intervals for 90 min.

### 2.8. Anti-Inflammatory Assay

Anti-inflammatory activity was performed as previously reported [18]. The murine peritoneal macrophage cells (RAW 267.4) were seeded in a 96-well plate at a density of 1 × 10^5^ cells per well and allowed to attach overnight. The spent medium was removed and replaced with 1 *μ*g/mL bacterial lipopolysaccharide (LPS) and five concentrations of the plant extract (6.5, 12.5, 25, 50, and 100 *μ*g/mL) or positive control, aminoguanidine (7 *μ*g/mL), and then incubated for 18 h at 37°C. To measure the nitric level, the spent medium (50 *μ*L) was removed and added to an equal volume of Griess reagent. The absorbance was then measured at 540 nm. Cell viability was determined using the MTT assay [19].

### 2.9. Immune-Stimulatory Potential

The RAW 267.4 macrophage cells were seeded in a 96-well plate at a density of 1 × 10^5^ cells per well and allowed to attach overnight. The spent medium was removed and replaced with varying concentrations (12.5, 25, 50, 100, and 200 *μ*g/mL) of the plant extract or positive control, bacterial lipopolysaccharide (1 *μ*g/mL), and then incubated for 18 h at 37°C. To measure the nitric level, 50 *μ*L of the spent medium was removed and added to the Griess reagent (50 *μ*L), and the absorbance was then observed at 540 nm.

### 2.10. Statistical Analysis

The data were statistically analysed by means of Student's *t*-test (two-tailed paired). Three replicates of each test sample (ethanol extract) were compared with three replicate values of the controls.

## 3. Results

### 3.1. Beta-Glucuronidase Activity

**Figure 1:**
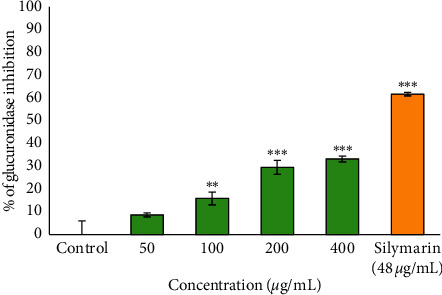
Inhibitory effect of the ethanol extract of *B. asphodeloides* on beta-glucuronidase activity. Data are presented as the mean ± SD (*n* = 3). *∗∗P* < 0.01 and *∗∗∗P* < 0.001 compared to the control.

The results of beta-glucuronidase inhibition are shown in Figure 1 which revealed significant inhibitory activity against the bacterial *ß*-glucuronidase enzyme in a dose-dependent manner with the greatest inhibition recorded at the concentration of 400 *μ*g/mL compared to the control. Silymarin, used as a positive control, demonstrated 61.7% inhibition of bacterial *ß*-glucuronidase activity.

### 3.2. Metal Ion Chelating Activity

**Figure 2:**
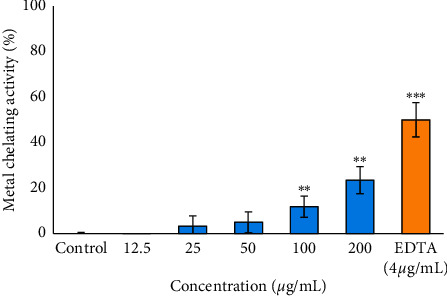
Inhibitory effect of the ethanol extract of *B. asphodeloides* on metal ion chelating activity. Data are presented as the mean ± SD (*n* = 3). *∗∗P* < 0.01 and *∗∗∗P* < 0.001 compared to the control.

To assess the preventive ability of the *B. asphodeloides* extract to limit chemical reactivity on the skin surface, inhibition of metal ion chelating activity was investigated. The result showed dose-dependent metal ion chelating activity (Figure 2); however, statistical significance was only reached at the higher concentrations (100 and 200 *μ*g/ml) as compared to the control, although weak relative to the positive control, EDTA.

### 3.3. Cytotoxicity

**Figure 3:**
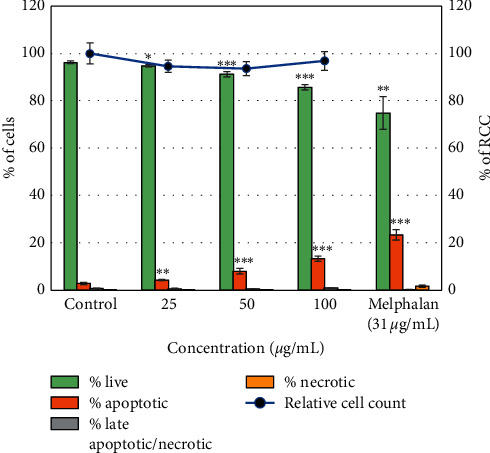
The cytotoxic effect of the *B. asphodeloides* ethanol leaf extract towards the human dermal fibroblast (MRHF). Data are presented as the mean ± SD (*n* = 3). *∗P* < 0.05, *∗∗P* < 0.01, and *∗∗∗P* < 0.001 compared to the control.

The cytotoxic effect of the *B. asphodeloides* extract towards MRHF cells was assessed using the Hoechst 33342/PI dual-staining method. Treatment of MRHF cells with the *B. asphodeloides* extract showed that the extract was not cytotoxic to the cell lines at the tested concentrations (Figure 3). Also, there was no significant (*P* < 0.001) cytotoxicity at all the tested concentrations as indicated by the percentage of live cells (decrease in the percentage of apoptotic cells as well as a significant increase in the relative cell counts). It was also evident that there was no significant (*P* < 0.001) increase in the percentage of late apoptosis/necrosis cells across all the tested concentrations (25–100 *μ*g/mL) as compared to the control cells. On the contrary, melphalan (31 *μ*g/mL), used as a positive control, exhibited a significant cytotoxicity (decrease in the percentage of live cells as well as increase in the percentage of apoptotic cells) compared to the trend seen in both the extract and the control.

### 3.4. Cellular Antioxidant Activity

**Figure 4:**
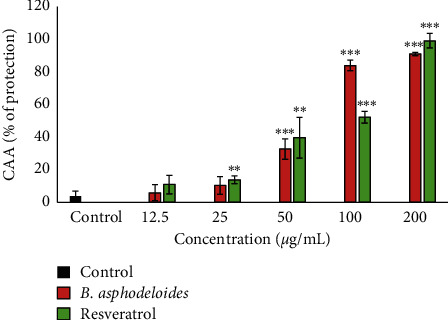
Cellular antioxidant activity of the ethanol extract of *B. asphodeloides*. Data are presented as the mean ± SD (*n* = 3). *∗∗P* < 0.01 and *∗∗∗P* < 0.001 compared to the control. CAA: cellular antioxidant assay.

The cellular antioxidant activities of *B. asphodeloides* are evaluated by the cellular antioxidant activity (CAA) assay using the MRHF cells. The degree of protection of the tested plant extract against the cellular conversion of DCF is illustrated in Figure 4. Included also is resveratrol, a known antioxidant compound. The results recorded indicated that the extract (12.5 *μ*g/mL–200 *μ*g/mL) exhibited significant antioxidant protection in a dose-dependent manner. At the highest tested concentration (200 *μ*g/mL), the effect of the extract was higher than that of the control, but less marked compared to the positive control, resveratrol.

### 3.5. Anti-Inflammatory Assay (NO Production by LPS-Activated RAW 264.7)

**Figure 5:**
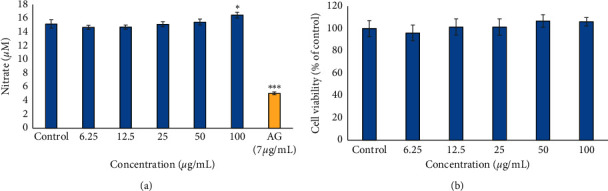
Effect of the ethanol extract of *B. asphodeloides* on (a) NO production in LPS-activated RAW 264.7 cells. (b) Corresponding cell viability is also shown. AG: aminoguanidine. Data are presented as the mean ± SD (*n* = 3). *∗P* < 0.05 and *∗∗∗P* < 0.001 compared to the control.

The result of the NO production by LPS-activated RAW 264.7 macrophages shows that the plant extract demonstrated no significant inhibition of NO production and noncytotoxicity towards activated RAW 264.7 at all the tested concentrations (6.25–100 *μ*g/mL) (Figure 5). By contrast, the positive control, aminoguanidine (7 *μ*g/mL), displayed better inhibition of NO production than the extract and the untreated control.

### 3.6. Immune-Stimulatory Potential

**Figure 6:**
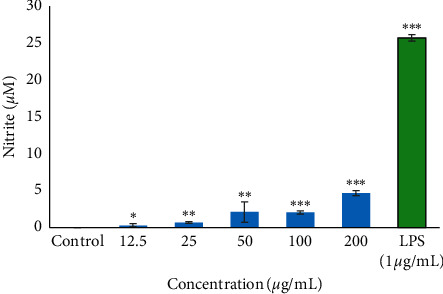
Immune-stimulatory potential of the ethanol extract of *B. asphodeloides* using RAW 264.7 cells. Previous experiment (anti-inflammatory assay) has confirmed the absence of toxicity. Data are presented as the mean ± SD (*n* = 3). *∗P* < 0.05, *∗∗P* < 0.01, and *∗∗∗P* < 0.001 compared to the control.

The result of the immune-stimulatory potential of the *B. asphodeloides* extract is shown in Figure 6. The result revealed that the extract exhibited no meaningful indication of immune-stimulatory activity. Although some of the higher concentrations revealed statistically significant levels of nitrite in the culture medium, these values are too low to be considered physiologically relevant. Lipopolysaccharides, used as the positive control, increased nitrite production in the culture medium as compared to both the extract and the control.

## 4. Discussion

*β*-Glucuronidase is a prominent exoenzyme responsible for unpleasant body odour. It plays a significant role by catalysing the hydrolysis of *β*-glucuronide conjugates of exogenous and endogeneous compounds in the body [20]. Inhibitors of this enzyme are thus considered for cosmetic products aiming to reduce body odour. In this study, the plant extract showed significant inhibition of bacterial *ß*-glucuronidase activity in a moderate dose-dependent manner. Report has shown that body odour can thus be combated by limiting the bacterial decomposition of apocrine sweat, either by preventing the sweat formation (antiperspirant), eliminating skin-resident bacteria, or by inhibiting the enzymes responsible for sweat decomposition [21]. To the best of our knowledge, this is the first study of the effect of the *B. asphodeloides* extract on the *ß*-glucuronidase enzyme, suggesting its possible use for antiodour applications.

Accumulation of heavy metals on the skin surface can deplete the stratum corneum antioxidant complement, mainly via Fenton chemistry, leading to oxidative stress and the induction of inflammation and barrier dysfunction. Strategies to avoid such effects include the chelation of metal ions to limit chemical reactivity on the skin surface. In this present study, the result of the metal ion chelating activity showed that the *B. asphodeloides* extract possesses iron chelation activity; however, this activity is relatively weak and only significant at high concentrations. It may however be argued that high concentration would be achievable on the surface of the skin, and thus, the extract may be expected (to some degree) to combat oxidative stress resulting from metal ion-catalysed reactive oxygen species (ROS) formation. Several previous studies have indicated that polyphenols are highly linked with metal chelating abilities due to the presence of hydroxyl and carboxyl groups attached to their structure [22–24]. Polyphenols, powerful metal chelating agents, act by inactivating metal ions by forming a complex with metals thereby preventing the metal ion from initiating lipid oxidation [25, 26]. It could be inferred from this study that the weak iron chelation activity exhibited by the plant extract may be due to the presence of polyphenolic compounds [13], which in turn chelate Fe^2+^ ions.

Medicinal herbs and their extracts are often erroneously perceived as safe. Therefore, in the evaluation of the therapeutic effects of medicinal plants, it is important to assess their cytotoxicity. The cytotoxicity assay was performed using the Hoechst 33342/PI dual-staining method, and the results indicated that the *B. asphodeloides* extract caused a significant decrease in the percentage of apoptotic cells as well as a significant increase in the RCC at all the tested concentrations, suggesting that the extract is not toxic towards MRHF cells. The cytotoxic effect of *B. asphodeloides* on MRHF cell lines has never been reported. However, studies have been conducted on the cytotoxicity of some *Bulbine* genus species. A study conducted by Pather [27] revealed that the leaf gel extract of *Bulbine frutescens* and *Bulbine natalensis* was not cytotoxic to human dermal fibroblast (MRHF) cells. This observation supports the finding obtained in this study. Therefore, it could be concluded from this study that *B. asphodeloides* may be potentially safe for users.

The cellular antioxidant assay (CAA) measures the net effect of both passive permeability and transporter-assisted permeation and therefore provides a more biologically relevant evaluation of the capacity for an antioxidant to accumulate within a cell. Antioxidants can only protect against oxidative stress if they can reach the environment where reactive oxygen species (ROS) is produced. In this study, the results observed revealed that the extract possesses antioxidant compounds that have cellular bioavailability and can function as an antioxidant within a cellular environment. This is the first study of the cellular antioxidant activity of *B. asphodeloides*, suggesting that the bioactive antioxidant in the plant extract was well engrossed into the MRHF cells thereby exhibiting antioxidant activity. It could be concluded from this study that the *B. asphodeloides* extract may be advantageous in obliterating oxidative damage instigated by free radicals which plays a major contributory role in skin diseases. Given that both the endpoint probe (DCF) and the initiator of oxidative stress (TBHP) used in this study do not occur naturally, further studies using a model in which both the stressor and endpoint measurement represent genuine biological/natural would be useful to confirm that the cellular antioxidant activity of the extract is indeed biologically relevant.

Nitric oxide (NO) overproduction involves a range of events, including the reaction of free radicals and oxidants associated with physiological disorders, such as acute and chronic inflammatory disease, which leads to tissue damage and organ dysfunction [24, 28]. In general, the result of this study showed that the *B. asphodeloides* extract displayed no inhibition of NO production in LPS-stimulated RAW cells. On the contrary, the extract does not display cytotoxicity towards RAW 264.7 cells. Interestingly, this corresponds to the result earlier reported showing that the extract was not toxic towards MRHF cells, which further confirms that the *B. asphodeloides* extract was not toxic to cells. Several previous studies have shown that most medicinal plants exhibit good anti-inflammatory effect [29, 30]. A report from Nguyen et al. [31] showed that the ethanol extract of *Lasia spinosa* effectively decreased NO production in LPS-stimulated RAW 264.7 cells thereby inhibiting inflammation. Another study conducted by Sagbo et al. [32] also indicated that the aqueous extract of *Brachylaena elliptica* reduced NO production in RAW macrophage cells thereby inhibiting anti-inflammatory activity. These results are in contrast to the findings observed in this present study, suggesting that *B. asphodeloides* might not be a good candidate for preventing skin inflammation.

Skin aging and diseases such as diabetes are strongly associated with a decline in immune competency, thereby contributing to skin conditions such as poor wound healing. The result of the immune-stimulating potential showed that the *B. asphodeloides* extract exhibited no indication of immune-stimulatory activity at all the tested concentrations; however, this does not rule out the possibility that the extract may have immune-boosting properties at other therapeutic targets.

## 5. Conclusion

The findings in this study indicated that the leaf extract of *B. asphodeloides* demonstrated no cytotoxic effect towards the MRHF and RAW 264.7 cells, thereby further supporting the safe use of this plant. The extract also possessed a significant cellular antioxidant, antiglucuronidase, and weak effect of metal chelating activities in a dose-dependent manner. However, the *B. asphodeloides* extract displayed no significant anti-inflammatory and immune-stimulatory activities. Taken together, the results of this present study showed that *B. asphodeloides* may be a useful therapeutic agent for the treatment of skin diseases, therefore supporting its ethnomedicinal usage. Further studies are warranted to isolate and characterize the active components of the plant extract.

## Data Availability

The data used to support the findings of this study are available from the corresponding author upon request.
